# Phosphoproteomic insights into processes influenced by the kinase-like protein DIA1/C3orf58

**DOI:** 10.7717/peerj.4599

**Published:** 2018-04-09

**Authors:** Agnieszka Hareza, Magda Bakun, Bianka Świderska, Małgorzata Dudkiewicz, Alicja Koscielny, Anna Bajur, Jacek Jaworski, Michał Dadlez, Krzysztof Pawłowski

**Affiliations:** 1 Department of Experimental Design and Bioinformatics, Faculty of Agriculture and Biology, Warsaw University of Life Sciences, Warszawa, Poland; 2 International Institute of Molecular and Cellular Biology, Warszawa, Poland; 3 Institute of Biochemistry and Biophysics, Polish Academy of Sciences, Warszawa, Poland; 4 Current affiliation: Max Planck Institute of Molecular Cell Biology and Genetics, Dresden, Germany; 5 Department of Translational Medicine, Clinical Sciences, Lund University, Lund, Sweden

**Keywords:** Phosphoproteomics, Novel kinases, Signalling, Secretory pathway, Mass spectrometry

## Abstract

Many kinases are still ‘orphans,’ which means knowledge about their substrates, and often also about the processes they regulate, is lacking. Here, DIA1/C3orf58, a member of a novel predicted kinase-like family, is shown to be present in the endoplasmic reticulum and to influence trafficking via the secretory pathway. Subsequently, DIA1 is subjected to phosphoproteomics analysis to cast light on its signalling pathways. A liquid chromatography–tandem mass spectrometry proteomic approach with phosphopeptide enrichment is applied to membrane fractions of DIA1-overexpressing and control HEK293T cells, and phosphosites dependent on the presence of DIA1 are elucidated. Most of these phosphosites belonged to CK2- and proline-directed kinase types. In parallel, the proteomics of proteins immunoprecipitated with DIA1 reported its probable interactors. This pilot study provides the basis for deeper studies of DIA1 signalling.

## Introduction

Protein kinase-like (PKL) proteins are a very large group of signalling and biosynthetic enzymes, that regulate most cellular processes, for example cell cycle, response to stimuli, and proliferation, by phosphorylating various substrates (Cohen, 2002) and are common in all branches of the tree of life (Kannan et al., 2007; Oruganty et al., 2016). The human genome encodes more than 500 protein kinases (Manning et al., 2002), and these proteins are among the most popular drug targets (Eglen & Reisine, 2011). According to the Pfam database, the PKL ‘clan,’ defined by sequence and structure similarities reflecting common evolutionary origin, includes 35 protein families (Finn et al., 2016). Nevertheless, this number is still growing, as more families are found to possess PKL three-dimensional fold, for example FAM20C (Tagliabracci et al., 2012) and COTH (Nguyen et al., 2016), or are predicted to be PKL-like (Dudkiewicz et al., 2012). The PKL proteins are characterised by a well-conserved structural scaffold, which carries a strongly conserved active site (Cheek et al., 2005; Taylor & Radzio-Andzelm, 1994), although within the PKL clan, sequence similarities between some families are rather low. Notwithstanding the high level of interest in kinases, research effort has been strongly biased (Edwards et al., 2011; Manning, 2009) with approximately 10% of known human kinases yielding at least 90% of kinase-related publications (Edwards et al., 2011). An important fraction (approximately 10%) of the PKL superfamily are proteins termed pseudokinases. These are homologues of kinases that have lost catalytic function but often have retained important roles in signalling, for example as allosteric activators of active kinases (Jacobsen & Murphy, 2017).

Although protein kinases were originally believed to act intracellularly, secretory kinases form an emerging novel addition to the PKL world (Sreelatha, Kinch & Tagliabracci, 2015; Tagliabracci et al., 2012, 2015). In humans, the kinase-like proteins related to the secretory pathway are likely far from being fully catalogued. The now established atypical protein kinases, FAM20s, localised in the Golgi apparatus, are secreted and phosphorylate secretory pathway proteins (Ishikawa et al., 2008; Koike et al., 2009; Tagliabracci et al., 2012). Nevertheless, FAM20C is not the only secretory kinase in bone, an organ where secretion is very important (Yang et al., 2016) which suggested that generally more secretory kinases may be at play in humans. Another secretory protein kinase, the PKDCC, was characterised earlier (Bordoli et al., 2014). Accordingly, using bioinformatic approaches, we previously predicted that a group of five uncharacterised human secreted proteins and their Metazoan homologues, the FAM69/DIA1 family (Aziz, Harrop & Bishop, 2011; Tennant-Eyles et al., 2011), are distant homologues of protein kinases (Dudkiewicz, Lenart & Pawlowski, 2013). With the exception of the pseudokinase DIA1R these proteins have well-conserved active site motifs and likely do have kinase activity (Dudkiewicz, Lenart & Pawlowski, 2013).

**Figure 1:**
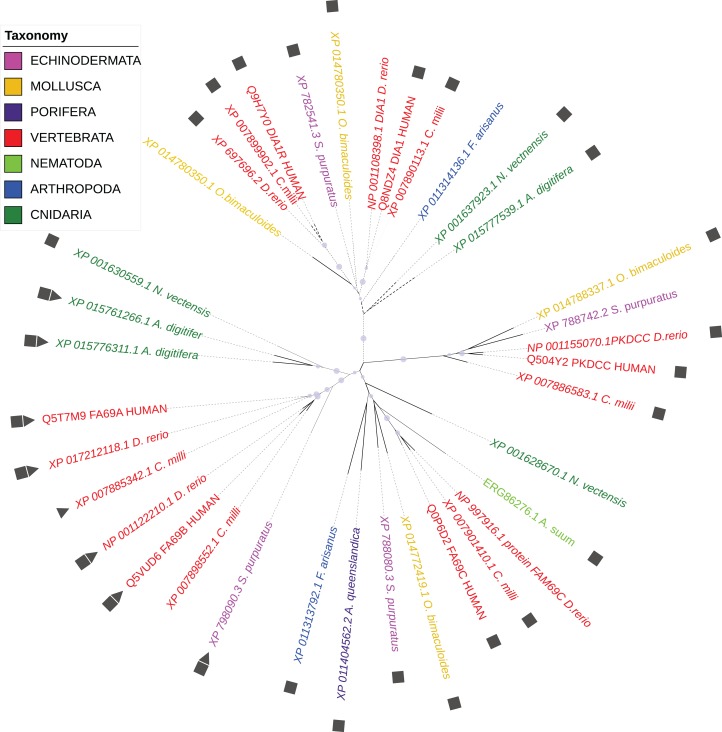
Phylogenetic tree of selected FAM69/DIA1 proteins. Colouring by taxonomy: red: vertebrates, pink: echinoderms, magenta: sponges, yellow: molluscs, light green: nematodes, dark green: cnidarians, blue: arthropods. Squares mark proteins predicted to be secreted, triangles—sequences with an ‘EF-hand’ calcium ion-binding motif inserted into the kinase domain. Circles mark branches with significant bootstrap values.

According to the WiKinome classification (WiKinome, 2017), FAM69/DIA1 proteins belong to Kinase Family RESK (REmote Secreted Kinases) that also contains the PKDCC secretory kinase (Bordoli et al., 2014; Maddala, Skiba & Rao, 2016). The FAM69/DIA1 family is ubiquitous in *Metazoa* and is already present in sponges. The family has three main branches (FAM69, DIA1 and PKDCC) that were probably present in the common ancestor of *Bilateria* and *Cnidaria* (see Fig. 1).

Here, we focus on DIA1, which is one of few uncharacterised predicted secretory kinases, and has been linked to autism (Morrow et al., 2008). The DIA1 protein (a.k.a. C3orf58, HASF, GoPro49, PIP49) has been previously shown to reside in the Golgi (Takatalo et al., 2008). DIA1 expression has been observed in cartilaginous mesenchymal tissues, regulated developmentally, with the highest expression seen in proliferating chondrocytes (Takatalo et al., 2008). Further, colocalisation with beta-coatomer protein has been observed, and is suggestive of a function in secretory processes (Takatalo et al., 2009). Then, characteristic expression of DIA1 has been observed in dental follicles, which may also suggest a role in secretion and trafficking (Takatalo et al., 2009). However, analysis of data deposited in the Allen Brain Atlas (Hawrylycz et al., 2012) indicates that DIA1 is also expressed in the brain tissue, including the hippocampus, olfactory bulb and cortex. Mass-spectrometry proteomic databases show relatively widespread expression of C3orf58 in the pancreas, adrenal gland, ovary and testis, lung, retina, placenta and platelets (Kim et al., 2014; Wilhelm et al., 2014).

The DIA1 protein has to date been studied experimentally without reference to the predicted kinase function. Evidence has been published for the role of DIA1 as a secreted paracrine factor in PKCε-mediated cytoprotection (Beigi et al., 2013; Huang et al., 2014; Miura, 2014), acting via interaction with the insulin-like growth factor 1 receptor (IGF1R) (Bareja et al., 2017). As discussed further below, these extracellular functions are quite intriguing, and may represent striking functional duality or growth-factor-like function for a kinase-like molecule.

Many uncharacterised proteins are ignored by biologists due to a lack of known or predicted molecular function (Pawlowski, 2008). Here, employing phosphoproteomics, a promising approach to elucidate effects of uncharacterised kinases (Amano et al., 2015), we present the first attempt to shed light on the molecular functions of one of the members of the novel FAM69 protein family in the context of the predicted kinase function. The main aims are to establish the secretory pathway localisation of DIA1 and to investigate the effects of its presence on membrane fraction phosphoproteome.

First, we verified the presence of DIA1 in the endoplasmic reticulum (ER) and Golgi, and using the RUSH system we provided evidence for a DIA1 role in secretory trafficking. Next, the phosphoproteome of membrane fractions of DIA1-overexpressing cells was compared with control cells with physiological levels of DIA1. We further investigated the interactome of DIA1. Finally, we related the phosphoproteomics results to protein functional relationship networks.

## Materials and Methods

### Antibodies

The following antibodies were used: rabbit anti-calnexin (2679; Cell Signaling Technology, Danvers, MA, USA), mouse anti-GM130 (610822; BD Biosciences, San Jose, CA, USA), mouse anti-E-cadherin (HECD-1 clone, 13-1700; ThermoFisher Scientific, Waltham, MA, USA), mouse anti-tubulin (T9026; Sigma-Aldrich, St. Louis, MO, USA), rabbit anti-histone 3 (H0164; Sigma-Aldrich, St. Louis, MO, USA), rabbit anti-DIA1/C3orf58 (ab103202; Abcam, Cambridge, UK), rat anti-HA (11867423001; Applied Science, Penzberg, Germany), anti-mouse, anti-rabbit, anti-rat antibodies conjugated with Alexa Fluor (Invitrogen, Carlsbad, CA, USA) or horseradish peroxidase (Jackson ImmunoResearch, West Grove, PA, USA).

### Plasmids

Plasmid encoding human *DIA1* cDNA was purchased from Source BioScience LifeSciences (IRAT Clone c3orf58; Nottingham, UK). pcDNA3-2HA plasmid was constructed based on pcDNA3 (ThermoFisher Scientific, Waltham, MA, USA) by inserting two repetitions of the HA sequence downstream of the multicloning site (a kind gift from Dr. Iwona Cymerman). pcDNA3Dia1HA was generated by subcloning PCR amplified human *c3orf58* cDNA using IRAT Clone c3orf58 as a template to *Bam*HI/*Eco*RI sites of pcDNA3-2HA. Str-li_VSVGwt-SBP-EGFP plasmid (a kind gift from Dr. Franck Perez, Boncompain et al., 2012; Addgene plasmid # 65300).

### In vitro cell line culture and transfection

HEK293T cells (ATCC, Manassas, VA, USA) and HeLa cells (Sigma-Aldrich, St. Louis, MO, USA) were grown under standard culture conditions (Swiech et al., 2011; Perycz et al., 2011). The cells were transfected using polyethylenimine (23966; Polysciences, Inc., Warrington, PA, USA) 24–48 h before any further analysis.

### Cell fractionation

Qproteome Cell Compartment kit (Qiagen, Redwood City, CA, USA) was used for subcellular fractionation. The collected fractions were analysed by standard Western blot protocol or by mass spectrometry.

### Live-cell imaging and image analysis

Twenty-four hours after transfection, HeLa cells growing on glass coverslips were transferred into a low profile open bath chamber (RC-41LP; Warner Instruments, Hamden, CT, USA) filled with culture media. At time 0, D-biotin (40 μM final concentration; Sigma-Aldrich, St. Louis, MO, USA) was added to cells. The time-lapse acquisition was performed at 37 °C and 5% CO_2_ atmosphere using spinning-disk microscope (Andor Revolutions XD, Belfast, UK) equipped with 63× oil objective and a thermostat-controlled chamber. Cell images were acquired as Z-stacks once per minute for 1 h at 1,004 × 1,002 pixel resolution and next converted using a maximum intensity projection function. For all the scans, microscope settings were kept the same. Image analysis was conducted with ImageJ software (National Institutes of Health, Bethesda, MD, USA). Time-lapse images were aligned with the StackReg plug-in for ImageJ (Thevenaz, Ruttimann & Unser, 1998). Next, three different regions of interest of the same size comprising the Golgi complex (the position of the Golgi complex was obtained using later time points) were selected and the total fluorescence intensity was measured for each time point using the Time Series Analyzer V3 plug-in for ImageJ and normalised to the maximum value (1). To assess statistical significance, the Mann–Whitney test was used.

### Immunofluorescence and imaging of immunofluorescently stained cells

For immunofluorescence staining, HeLa cells were fixed and immunofluorescently stained as described (Swiech et al., 2011). Images of stained cells were obtained using a Zeiss NLO 710 confocal microscope with a 40× oil objective (1,024 × 1,024 pixel resolution) as Z-stacks of the images (averaged twice per line) and next converted to single images with a maximum intensity projection function.

### Immunoprecipitation

Twenty microliters of Pierce anti-HA Magnetic Beads (ThermoFisher Scientific, Waltham, MA, USA) were rinsed with lysis buffer (150 mM KCl, 20 mM HEPES–NaOH (pH 7.5), 0.3% CHAPS, protease and phosphatase inhibitors). Beads were incubated with cell lysate (800 μl, obtained from four million cells) from DIA1-HA expressing cells and from control cells for 45 min at 4 °C, and next were washed three times using 300 mM NaCl, 0.1% CHAPS, 20 mM HEPES–NaOH, pH 7.5 and finally resuspended in 25 mM HEPES (pH 7.5), 1 mM MnCl_2_, 10 mM MgCl_2_, 1 mM CaCl_2_, and 50 mM KCl. The experiment was repeated twice in pH 5 and twice in pH 7, thus four DIA1 and four control samples were available for liquid chromatography–tandem mass spectrometry (LC-MS/MS) experiments.

### Proteomic sample preparation

For protein extraction, the methanol/chloroform method was used (Wessel & Flugge, 1984). Pellets were diluted in 6 μl 15% sodium deoxycholate and then 100 mM NH_4_HCO_3_ was added to obtain a final volume of 86 μl. TCEP (0.5 M) was added to a sample for a final concentration of 5 mM and incubated at 60 °C for 1 h. To block reduced cysteine residues, 200 mM MMTS at a final concentration of 10 mM was added and the sample was subjected to incubation at room temperature for 10 min. For digestion, trypsin (Promega, Madison, WI, USA) was added at a 1:20 v/v ratio and the sample was incubated at 37 °C overnight. Finally, trifluoroacetic acid was used to inactivate trypsin and remove sodium deoxycholate. Resulting peptides were analysed by LC-MS/MS for peptide identification or phosphopeptide enrichment.

### Phosphopeptide enrichment with Ti-IMAC

For phosphopeptide enrichment, MagReSyn Ti-IMAC was used according to the Ti-IMAC manufacturer’s protocol.

### LC-MS/MS settings

Liquid chromatography–tandem mass spectrometry proteomic analyses of samples were performed with an LTQ-Orbitrap Elite mass spectrometer (ThermoFisher Scientific, Waltham, MA, USA) coupled with a nanoACQUITY (Waters Corporation, Milford, MA, USA) UPLC system. Measurements were conducted in positive polarity mode, with capillary voltage set to 2.5 kV. A sample was initially applied to the nanoACQUITY UPLC Trapping Column (Waters) while water containing 0.1% formic acid was used as a mobile phase. Then, the peptide mixture was transferred to a nanoACQUITY UPLC BEH C18 Column (75 μm inner diameter; 250 mm long; Waters), applying an acetonitrile gradient (5–35% acetonitrile over 160 min) in the presence of 0.1% formic acid with a flow rate of 250 nl/min. Peptides were eluted directly to the ion source of the mass spectrometer. Before each LC run, a blank run was performed to ensure no material was carried-over from a previous analysis.

Higher-energy Collisional Dissociation (HCD) fragmentation was applied. Up to 10 MS/MS events were allowed per MS scan. See the Supplemental Information for additional MS analysis parameters.

### Qualitative MS data processing and database search

The raw data files acquired by MS and MS/MS were processed using MaxQuant software (version 1.5.8.3). The ion lists were searched against the SwissProt human database (20,274 sequences) using the Andromeda search engine (Cox et al., 2011). The search parameters were set as follows: enzyme—trypsin; fixed modifications—cysteine modification by MMTS; variable modifications—oxidation of methionine, phosphorylation of serine, threonine and tyrosine. Peptide mass tolerance was set to 40 ppm for recalibration and 5 ppm for the main search. The ‘match between runs’ option was used to transfer identifications to other LC-MS runs based on their masses and retention time with a maximum deviation of 0.7 min. Both proteins and peptides were identified with a false discovery rate (FDR) of 1%. The MaxQuant label-free algorithm was used for protein quantification in non-phospho-enriched samples.

### Proteomics data

The mass spectrometry proteomics data have been deposited with the ProteomeXchange Consortium via the PRIDE (Vizcaino et al., 2016) partner repository with the dataset identifier PXD006831.

### Bioinformatics

Statistical analysis of differences in proteome and phosphorylation state between six WT and six DIA1-transfected samples was performed in Perseus software (version 1.5.8.5) (Tyanova et al. 2016). MaxLFQ intensities of protein groups in non-phospho-enriched experiments and phosphosite intensities in enriched samples were further processed excluding peptide matches from decoy and contaminant databases. Additionally, for phospho-enriched samples, intensities of phosphorylation sites were subjected to statistical analysis if the localisation probability exceed 75%. For non-enriched membrane samples, proteins identified only by modification site were deleted from analysis. All values for each replicate were log2 transformed and normalised by median subtraction. Quantitative analysis was performed using the *t*-test with permutation-based FDR correction only for PTM sites or proteins identified in at least four out of six replicates in each group. Those differences with a FDR *q*-value less than 0.05 were considered statistically significant. Also, hits were selected as qualitative changes, if they were quantified in at least four samples of the six samples in one group and not detected in the other sample set.

For analysis of DIA1 interactors, four replicates from control and DIA1 groups were compared using the *t*-test in a similar manner as for non-phospho-enriched samples. In the interactome analysis, protein groups were subjected to quantitative analysis with the *t*-test if they were quantified in at least three samples in each group. Also, proteins were considered to significantly differ in abundance qualitatively, if they were quantified in at least three samples out of four in one group and not detected in the other sample set.

Functional analysis of lists of proteins was performed using the DAVID and Panther servers (Huang, Sherman & Lempicki, 2009; Mi et al., 2013). Overrepresentation of specific functional annotations within the lists was determined by Fisher’s exact test as described previously (Welinder et al., 2017). Gene Ontology annotations, SwissProt keywords, and KEGG pathways were used as annotation terms for the enrichment analysis. For lists of proteins with significantly changed expression, the background protein sets consisted of all proteins detected. For visualisations, the Spotfire program was used (Tibco Software, Inc., Palo Alto, CA, USA).

Further functional analysis of the regulated phosphoproteins was conducted using Ingenuity Pathway Analysis (IPA) (Qiagen, Redwood City, CA, USA), in particular by generating networks of protein–protein relationships. As background, the set of all proteins detected in the experiment was used.

The KinomeXplorer toolset (Horn et al., 2014; Linding et al., 2008; Miller et al., 2008) was applied to predict likely kinases causing the observed phosphorylation changes. Two programs were used, NetPhorest, the atlas of consensus phosphorylation site sequence motifs, and NetworKin, which adds protein interaction information to improve NetPhorest predictions.

The PhosphoSitePlus database (Hornbeck et al., 2012, 2015) was used to relate the observed phosphosites to literature findings.

The GibbsCluster-2.0 Server was applied for clustering of phosphosite sequences. The regions surrounding the phosphorylated residue were considered (plus–minus three residues), and several desired numbers of clusters were tested (Andreatta, Alvarez & Nielsen, 2017).

For the phylogenetic tree, homologues of human FAM69A-C, DIA1, DIA1R and PKDCC proteins were identified for seven different taxonomy groups using classic blastp searches and multiple sequence alignment was built using the Promals3D server (Pei, Kim & Grishin, 2008). A maximum likelihood phylogenetic tree was constructed for whole aligned sequences, with the aLRT statistical test for branch support using the phyML method implemented in the phylogeny.fr server (Dereeper et al., 2008). EF-hand motifs in analysed sequences were identified using the ScanProsite tool (Gattiker, Gasteiger & Bairoch, 2002) while signal peptides were predicted using the SignalP program 3.0 (Bendtsen et al., 2004).

## Results and Discussion

### Analysis of secretory traffic kinetics in DIA1-expressing living cells

**Figure 2:**
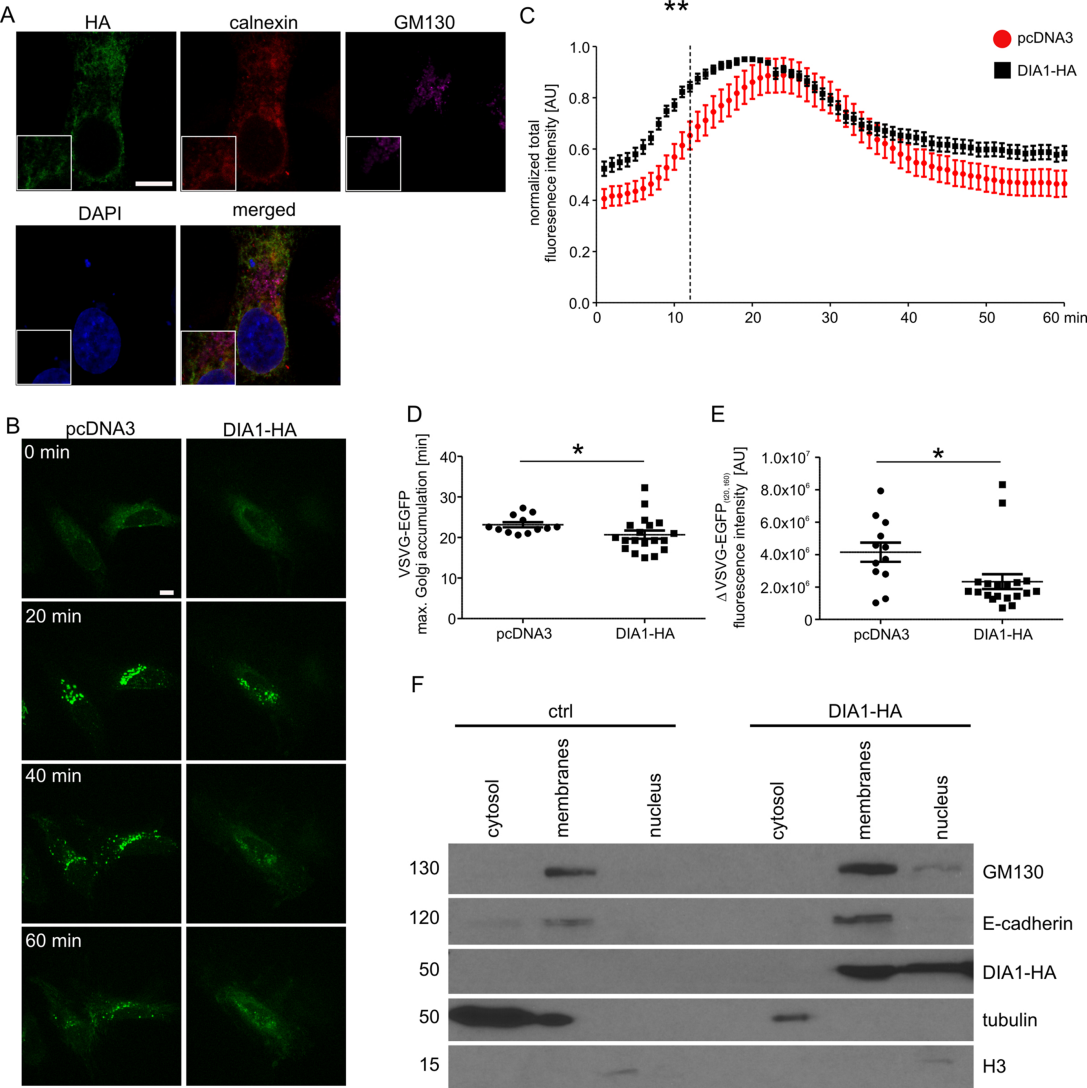
(A) Representative confocal images of HeLa cells transfected with pcDNA3Dia1HA and immunofluorescently stained for HA tag (green), ER marker calnexin (red) and cis-Golgi marker GM130 (magenta). Cells were counterstained with Hoechst 33285 to visualise cell nuclei. Inlets show zoomed in areas containing parts of ER and Golgi. Scale bar = 10 μm. (B) Representative time-lapse confocal images of living HeLa cells transfected with Str-li_VSVGwt-SBP-EGFP and DIA1-HA or pcDNA3 vector as a control, in which secretory trafficking of the fluorescent reporter protein (VSVG–EGFP) was analysed using the RUSH system for 60 min after biotin addition (time 0). Scale bar = 10 μm. (C) Quantitative analysis of the live imaging experiments performed as in (B). The plot shows VSVG–EGFP fluorescence intensity in the Golgi region at each time point normalised to the maximum value. The data are expressed as mean for all analysed cells. Error bars indicate SEM. **p < 0.01 (Mann–Whitney test). Number of independent experiments *N* = 3. Number of cells per variant (*n*): pcDNA3 (12), DIA1-HA (19). (D) Graph shows a comparison of VSVG–EGFP maximum accumulation times in the Golgi area. The data are expressed as mean for all analysed cells. Error bars indicate SEM. *p < 0.05 (Mann–Whitney test). Number of independent experiments and analysed cells per variant as in (C). (E) Graph shows a comparison of VSVG–EGFP protein fractions accumulated in the Golgi complex (time = 20 min) that reached the plasma membrane at time = 60 min (expressed as *t*_20_–*t*_60_ min). The data are expressed as mean for all analysed cells. Error bars indicate SEM. *p < 0.05 (Mann–Whitney test). The number of independent experiments and analysed cells per variant as in (C). (F) Representative images of Western blot analysis of HEK293T cell fractions from control cells and cells transfected with DIA1-HA. GM130, E-cadherin served as a marker of the membrane fraction while alpha tubulin and histone 3 (H3) served as markers of cytosolic and nuclear fractions, respectively (left panel). The fractionation experiment was repeated three times.

Previous work has shown that DIA1 resides in Golgi but its functional role there has not been analysed. Therefore, we decided to address the question of the potential role of DIA1 in cargo trafficking via ER–Golgi apparatus to the plasma membrane. First, however, we confirmed that DIA1 is indeed present in the secretory pathway. Toward this end, HeLa cell line was transiently transfected with a DIA1-HA plasmid encoding DIA1 tagged on its C-terminus with a double HA tag and immunofluorescence staining was performed for the HA tag and calnexin (an ER marker) and GM130 (a cis-Golgi marker). As shown in Fig. 2A, in HeLa cells DIA1-HA was mostly located in ER. Thus, we compared synchronised trafficking of cargo in control and DIA1-overexpressing HeLa cells. We used the RUSH system (Boncompain et al., 2012) using VSVG–EGFP as a reporter. This system contains two major elements that need to be introduced to cells: (i) streptavidin fused to resident ER protein (Ii; so called hook) and (ii) a reporter connected to streptavidin binding peptide. In transfected cells, biotin treatment leads to release from the hook and synchronised trafficking of the reporter (Boncompain et al., 2012). Thus, we tracked the VSVG–EGFP trafficking in living cells within 60 min of biotin addition at time 0 (Fig. 2B). Image analysis revealed that both in control and DIA1-expressing cells there was an accumulation of VSVG–EGFP in the Golgi complex but dynamics of this process was different (Fig. 2C). In DIA1-overexpressing cells the maximum accumulation in the Golgi complex occurred 4 min faster than in the control (Fig. 2D). We also observed no disturbances in reaching the plasma membrane for DIA1-overexpressing cells and control cells. Nevertheless, the fraction of reporter protein accumulated in the Golgi complex at 20 min that reached the plasma membrane at 60 min was lower for Dia1-overexpressing cells (Fig. 2E). The obtained results show that in HeLa cells, DIA1 is present in elements of the secretory pathway (ER) and its increased abundance in the ER accelerates the transition of secretory cargo from the ER to Golgi and slows down its release from this compartment. A previous study by Takatalo et al. (2009) showed the presence of DIA1 primarily in Golgi, while Bareja et al. (2017) claimed DIA1 extracellular protein. These findings are not necessarily contradictory and suggest that DIA1 is present through the whole secretory pathway to be finally secreted. The observed differences likely reflect differences between cell lines and constructs used in these studies. However, a question arises as to how DIA1 affects secretory trafficking. Thus far, a biological function has been assigned only to extracellular DIA1 (i.e. modulation of IGF1R activity; Bareja et al., 2017) and one possibility is that DIA1 affects ER–Golgi–PM trafficking acting on IGFR1. In fact, it has previously been shown that IGF-1 accelerates secretion of chromogranin A via Arf1, an GTPase controlling Golgi trafficking (Münzberg et al., 2015). This would explain why DIA1 affects not only ER–Golgi trafficking, but also cargo release from Golgi (where it is less abundant in HeLa cells). On the other hand, as we show below, DIA1 interacts with many ER and Golgi proteins, and such interactions could be involved in the regulation of cargo trafficking. In such case, intra- and extracellular functions of DIA1 would be different.

### Phosphoproteome of membrane fraction of WT and DIA1-expressing HEK293T cells

Because DIA1 is present in the secretory pathway and there potentially plays there a functional role, we decided to compare phosphoproteomes of isolated membrane fractions from control and DIA1-overexpressing HEK293T cells. We transiently transfected cells with a DIA1-HA plasmid and performed cell fractionation. As shown in Fig. 2F, the obtained fractions were relatively pure and DIA1-HA was present in the fraction containing cellular membranes. It should be noted however, that in DIA1-overexpressing HEK293T cells, DIA1-HA was also present in the nuclear fraction. This may reflect differences between cell lines or it may stem from partial contamination of nuclear fraction with the membrane fraction. Yet, in our opinion DIA1-HA presence in nuclear fraction should not directly affect the phosphoproteomics analysis performed on membrane proteins. After confirming the purity of the obtained membrane fractions and effective overexpression of DIA1-HA, membranes from HEK293T cells, control and overexpressing DIA1-HA were subjected to phosphoproteomic analysis. Figure S1 shows heat maps of Pearson correlation coefficient and multiscatter plots demonstrating the reproducibility of samples in proteome and phosphoproteome of membrane fractions. By applying conservative proteomics criteria, 2,758 protein groups (20,147 peptides, 563 phosphosites) were detected in the membrane fraction for the control cells, and 2,265 protein groups (20,051 peptides, 620 phosphosites) in the DIA1-HA overexpressing ones. After Ti-IMAC phosphopeptide enrichment, 2,030 protein groups (6,086 peptides, 2,752 phosphosites) were measured in the control cells, and 1,770 protein groups (4,518 peptides, 2,757 phosphosites) in the DIA1-HA overexpressing cells. The number of phosphorylated and non-phosphorylated proteins in the membrane fraction and after Ti-IMAC phosphopeptide enrichment is illustrated in Fig. S2. By merging the pre-enrichment and enriched result sets, 3,551 protein groups were identified in the control cells and 2,534 in the DIA1-HA overexpressing ones. A total of 3,764 protein groups were detected in 12 samples of the membrane fraction (six control samples and six DIA1-HA samples), represented by 22,647 peptides with 6,921 phosphosites. A total of 1,920 phosphosites in phospho-enriched samples satisfied the criterion of being quantified in at least four samples in both groups (DIA1 and WT). Additional information on the phosphosite statistics in enriched samples are provided in the Supplemental Information file.

**Figure 3:**
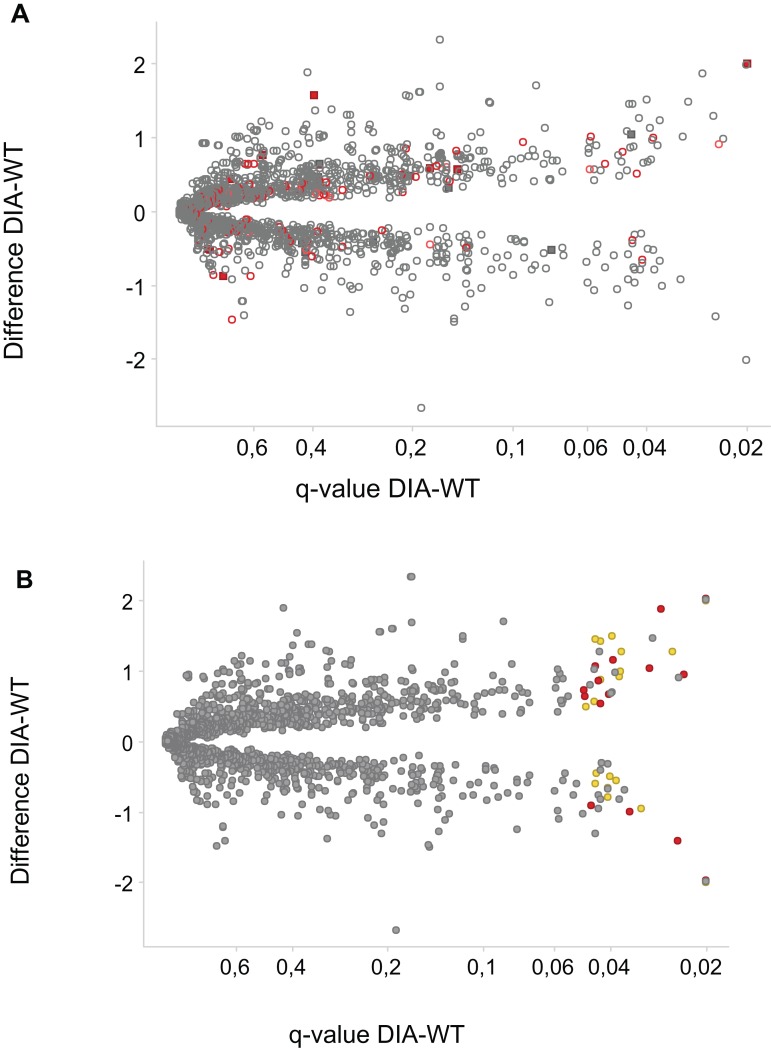
Phosphosites affected by DIA1 expression. Difference WT-DIA1 (average signal intensity for DIA1 vs. average intensity for WT samples; *y* axis) plotted against *t*-test *q*-value (*x* axis). Data are in log2 scale: hence, difference of 1 corresponds to a two-fold change. (A) Red: phosphosites within proteins annotated as ER or Golgi-resident. Filled squares: proteins possessing signal peptides. (B) Red: phosphosites predicted to be CK2 substrates. Yellow: phosphosites predicted to be CDK substrates.

Assessment of changes in phosphopeptide abundance yielded 64 significantly changed phosphosites in 56 proteins. Eight of the phosphosites on eight proteins were threonine sites, and the rest—serine sites. The numbers of changed phosphosites are of the similar order as in a recent paper studying phosphoproteomics of processes regulated by the LRRK2 kinase (Steger et al., 2016). The majority of the changed phosphosites have increased abundance in the DIA1-overexpressing cells (Fig. 3). The effects of DIA1 expression on phosphorylation are widespread, and many affected proteins are apparently not in the secretory pathway, i.e. do not possess a signal peptide and are not annotated as ER or Golgi proteins (Fig. 3). Thus, apparently DIA1 presence causes a widespread response, likely due to signalling beyond its immediate cellular compartment.

**Figure 4:**
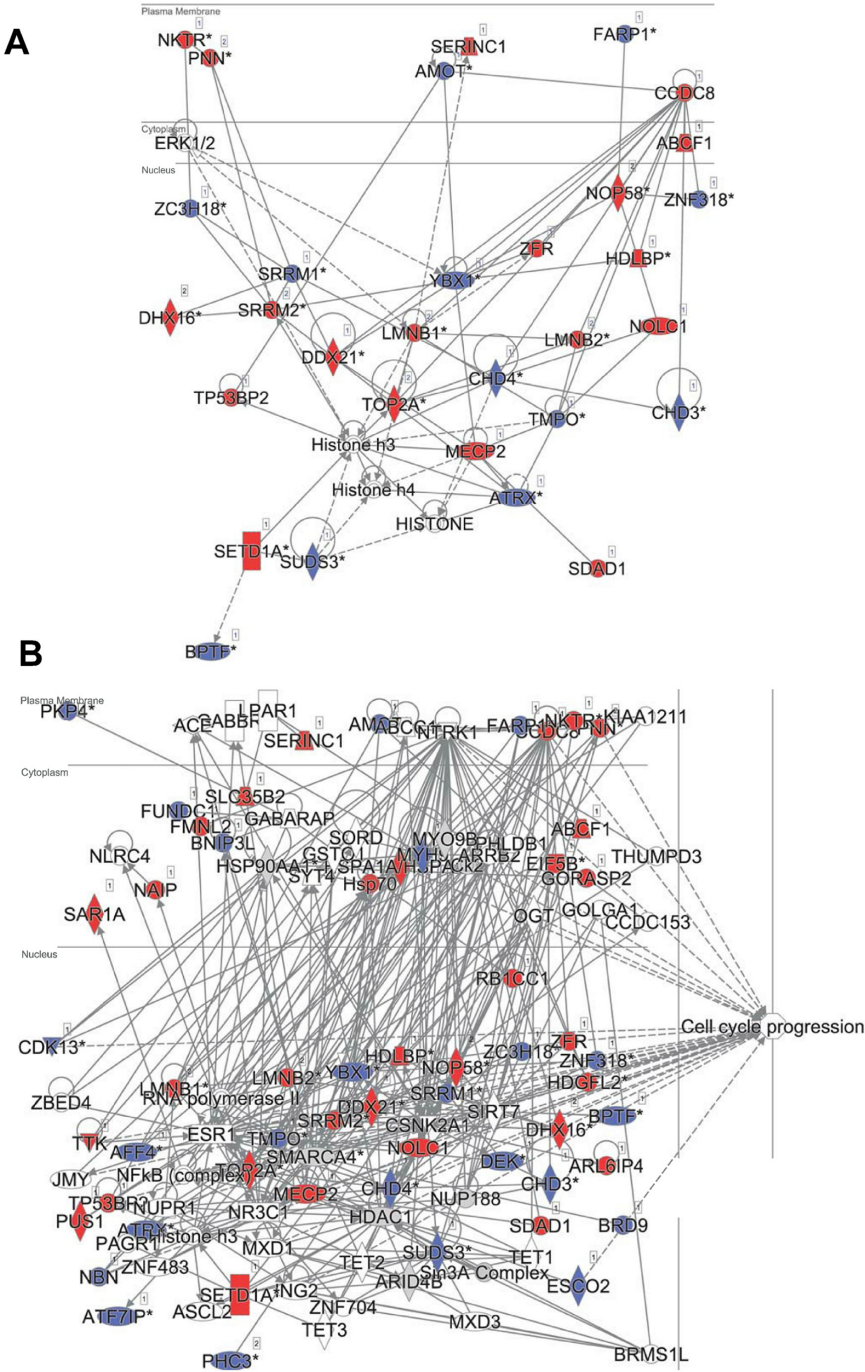
Ingenuity IPA analysis top subnetworks. Functional relationships between phosphoproteins affected by DIA1 expression and tightly related proteins. Red: phosphoproteins with phosphosite abundance increased upon DIA1 expression. Blue: phosphoproteins with phosphosite abundance decreased. (A) First subnetwork in IPA analysis using direct and indirect relationships. (B) Merged subnetworks 1, 2 and 3 in IPA analysis using only direct relationships. Proteins related to cell cycle marked.

In principle, an observed change in abundance of a phosphosite can be the result of change in abundance of the given protein. To test whether this was not the case, we examined the set of proteins whose abundance was significantly affected by DIA1. Indeed, none of these proteins matched the set of proteins with changed phosphosites with the only one obvious exception, DIA1 itself. According to the mass spectrometry analysis, in DIA1-overexpressing cells the levels of this protein increased more than 2,000-fold. Otherwise, only three other proteins had expression levels changed in response to DIA1 overexpression: Nucleolar transcription factor 1 (UBTF), 78 kDa glucose-regulated protein (HSPA5) and pre-mRNA-splicing factor CWC22 (see Supplemental Information). The two nuclear proteins, CWC22 and UBTF, were downregulated by DIA1 while the ER chaperone HSPA5 was upregulated. This suggests that some of the DIA1 functions in the secretory pathway may be exerted indirectly, by regulating the expression of this chaperone. The set of proteins whose phosphorylation status was significantly affected by DIA1 overexpression was subjected to the IPA system. IPA Core analysis (Fig. 4) maps the query set of proteins onto a literature-derived network of protein relationships and extracts subnetworks enriched in the query proteins. Thus, it can extract functional modules enriched in a protein set. In the case of phosphoproteins dependent on DIA1 expression, notable is the widespread effect, involving many nuclear proteins as well as some cytoplasmic organelle and plasma membrane proteins (Fig. 4). On one hand, it could be explained by the fact that the membrane fraction contains diverse membranes besides Golgi and ER and some proteins can reside outside their prototypical cellular location. For example several nuclear proteins, which we found in the membrane fraction phosphoproteome, were previously reported not only in the nucleus but also in membranous organelles called exosomes. Secondly, this observation may reflect the broad cellular effects of DIA1. Finally, the biochemical fractionation never provides 100% pure fractions and such ‘contaminations’ could be identified with such sensitive method as mass spectrometry. Here, for the set of phosphoproteins affected by DIA1 overexpression, the top subnetworks were enriched in molecules involved in Cancer, Cell Morphology, Cellular Assembly and Organization. In agreement with reported role of DIA1 in stimulating cell cycle progression (Beigi et al., 2013), many of the network proteins are involved in cell cycle (see Fig. 4).

In the IPA analysis, among the general functional annotations overrepresented in the query protein set, Cell Morphology, Cellular Assembly and Organization, and Cellular Development were most significant. These changes in the cell phosphoproteome agree with the proliferation and cell cycle effects of DIA1 observed by Dzau and co-workers in cardiomyocytes (Beigi et al., 2013; Bareja et al., 2017).

An analysis of functional annotations overrepresented among proteins with phosphosites affected by DIA1 expression was also performed using the Panther and DAVID servers. The conclusions provided by the two servers were similar: namely, very few overrepresented functional annotations were assigned to the query protein set, which highlights the diversity of the processes affected by DIA1 expression.

**Figure 5:**
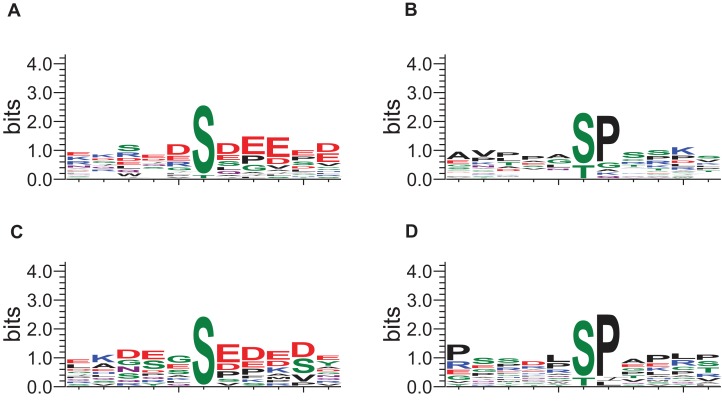
Sequence logos for phosphosites affected by DIA1 expression (GibbsCluster results). (A, C) CK2-like sites; (B, D) proline-directed kinase sites. (A, B) Sites upregulated in DIA1-overexpressing cells. (C, D) Sites downregulated in DIA1-overexpressing cells.

The local sequence motifs surrounding the phosphosites affected by DIA1 expression were submitted to the GibbsCluster server to elucidate the most common motifs. The DIA1-dependent phosphosites were clearly grouped into two distinct sequence clusters: 25 sites resembled the S[DE], Sx[DE] or Sxx[DE] consensus reminiscent of the CK2 site, while 29 phosphosites (see Fig. 5) matched the [ST]P consensus reminiscent of proline-directed kinases, for example CDK family, MAPK family and GSK3. Clearly, precise assigning the changed phosphosites to particular kinases and elucidating kinase-specific substrate motifs is challenging and may require specific tools as specific inhibitors or knock-out mice (Steger et al., 2016) that are not readily available for a novel poorly studied kinase-like protein such as DIA1. In order to attempt mapping of the differential phosphosites to known kinase substrate sites, the KinomeXplorer server was used. Notably, both prediction tools (NetworKin and NetPhorest) predicted that a substantial number of affected sites are putative CK2 sites (see Fig. 3B). Also, the sites predicted as CK2-like obtained higher confidence scores than the other sites. However, it has to be noted that the challenging task of assigning phosphosites to kinases is difficult because the datasets and tools available may be biased towards common motifs and well-studied kinases.

The DIA1 protein itself is found to be phosphorylated on five residues, S124, S191, S378, S387 and S426, none of which can be found in the PhosphositePlus database, where this protein is shown to be phosphorylated on four other sites: S13, Y92, Y96 and Y183. The functional role of phosphorylation on S124, S191, S378, S387 and S426 is not clear, although three of these residues (S124, 387 and 426) are not conserved evolutionarily in DIA1 homologues, and therefore may be not important in the regulation of DIA1 function. Also, some of these sites occur in DIA1 regions for which structure prediction is difficult. S124 and S191 are located in the kinase N-lobe, in a region corresponding to Ca^2+^-binding EF-hand motif insertion seen in FAM69A and FAM69B. S124 is part of a QSIC motif, where it can be speculated that phosphorylation affects the disulfide-bonding capability of the cysteine C125, and in consequence, the relative arrangement of the N- and C-lobes of the kinase domain. The S378, S387 and S426 are located in alpha-helical regions of the C-terminal lobe, likely far from the predicted catalytic site. Considering the spatial relation to the predicted active site, the two conserved phosphosites in DIA1 (S191 and S378) are more likely to affect interactions with binding partners (e.g. substrates) than catalysis itself. However, it is not completely unlikely that phosphorylation, possibly coupled with disulfide formation by some of the highly conserved Cys residues (Dudkiewicz, Lenart & Pawlowski, 2013), regulates DIA1 kinase activity.

A number of phosphosites affected by the presence of DIA1 occurred in proteins located in the ER or Golgi or otherwise related to the secretory pathway and were subject to further scrutiny.

### Interactome of DIA1

In this complementary analysis, we wanted to find potential DIA1-interacting proteins. Thus eluates from beads exposed to extracts from DIA1-HA-expressing HEK293T cells (four samples) were compared by LC-MS/MS proteomics with those from control cells (four samples). In total, 265 protein groups were measured in the eluates as being present in at least three samples in each group, and a *t*-test showed that 16 proteins were likely DIA1 binders. A further seven proteins were likely DIA1 binders since they were detected in at least three DIA1 samples and absent from all control samples. Of these 23 proteins, only three proteins were also identified as having phosphorylation status dependent on DIA1 presence: ARL6IP4, YBX1 and SRRM2. These may be considered potential substrates, if DIA1 is indeed proven to be a functional kinase. However, such a conclusion is speculative at this stage, because these are mostly nuclear proteins unlikely to interact with DIA1 directly and also because they have very diverse phosphosites (SxxE, SE and SP, respectively, see Table 1). Notably, this may also reflect that upon overexpression of DIA1-HA in HEK cells, some overexpressed protein could be found in the nuclear fraction and act there. Of the potential DIA1 interactors, 11 also had Golgi or ER localisation, including GIGYF2 (GRB10-interacting GYF protein 2), SEC61B (protein transport protein Sec61 subunit beta), and LRRC59 (leucine-rich repeat-containing protein 59). The latter 11 proteins may be considered likely physiological interactors of DIA1 and may be together involved in regulation of cargo trafficking.

**Table 1:** Selected phosphosites observed in this study as being dependent on DIA1 presence and related to the secretory pathway or otherwise related to the predicted DIA1 kinase activity.

Protein ID	Protein name	Gene name	Phosphosite motif	Phosphosite position	Student’s *T*-test *p*-value DIA_WT	Student’s *T*-test *q*-value DIA_WT	DIA1 vs. WT FC	Localisation
Q9H0W5	Coiled-coil domain-containing protein 8	CCDC8	PQASPRR	261	NA	NA	UP	Cytoskeleton, cytoplasm
Q59H18	Serine/threonine-protein kinase TNNI3K	TNNI3K	EVFTQCT	637	NA	NA	UP	Nucleus, cytoplasm
Q9H307	Pinin	PNN	QQDSQPE	381	0.0004	0.028	3.7	Nucleus
Q9NR31	GTP-binding protein SAR1a	SAR1A	IDRTDAI	139	0.0018	0.045	2.1	ER/Golgi
Q9H8Y8	Golgi reassembly-stacking protein 2	GORASP2	MAGTPIT	222	0.0014	0.038	2.0	Golgi
Q66PJ3	ADP-ribosylation factor-like protein 6-interacting protein 4	ARL6IP4	WHRSAGE	332	0.0001	0.024	1.97	Nucleus; DIA1 interactor
P0DMV9	Heat shock 70 kDa protein 1B	HSPA1B	KGGSGSG	631	0.0014	0.039	2.0	Nucleus, cytoplasm, ER
Q8TB61	Adenosine 3′-phospho 5′-phosphosulfate transporter 1	SLC35B2	PVESPVQ	427	0.0004	0.024	1.9	Golgi
Q8TDY2	RB1-inducible coiled-coil protein 1	RB1CC1	RLDSLPE	222	0.0022	0.046	1.7	Cytoplasm, ER
P33981	Dual specificity protein kinase TTK	TTK	LLNSPDC	281	0.0008	0.039	1.6	Cytoskeleton
Q9H307	Pinin	PNN	RQESDPE	100	0.0003	0.048	1.6	Nucleus
*Q8WVM8*	*Sec1 family domain-containing protein 1*	*SCFD1*	*VENSPAG*	*303*	*0.0212*	*0.156*	*1.5*	*ER/Golgi*
*P27824*	*Calnexin*	*CANX*	*GTVSQEE*	*564*	*0.0310*	*0.179*	*1.49*	*ER*
*P53621*	*Coatomer subunit alpha*	*COPA*	*KNLSPGA*	*173*	*0.0037*	*0.058*	*1.5*	*Golgi*
Q9NRX5	Serine incorporator 1	SERINC1	SDGSLED	364	0.0012	0.042	1.4	ER
Q14004	Cyclin-dependent kinase 13	CDK13	GDVSPSP	383	0.0011	0.043	0.8	Nucleus, cytoplasm, Golgi
Q56NI9	*N*-acetyltransferase ESCO2	ESCO2	NQGSPFK	75	0.0010	0.041	0.6	Golgi, nucleus
O60238	BCL2/adenovirus E1B 19 kDa protein-interacting protein 3-like	BNIP3L	SSQSEEE	120	0.0021	0.046	0.53	Nucleus, ER, mitochondrion
Q9UQ35	Serine/arginine repetitive matrix protein 2	SRRM2	LSYSPVE	2694	0.0004	0.032	0.5	Nucleus; DIA1 interactor
P67809	Nuclease-sensitive element-binding protein 1	YBX1	NEGSESA	174	NA	NA	DOWN	Nucleus, cytosol; DIA1 interactor

**Notes:**

All these phosphosites (except the one in TNNI3K) are listed in the PhosphositePlus database. For comparative analysis of all phosphosites, see the Supplemental Information. Note that for some phosphosites the *t*-test was not feasible due to their not being detected in one of the compared conditions (DIA1 or WT). Phosphosites whose difference between DIA1 and WT was not strictly significant are shown in italics. Full results are provided as Supplemental Information.

### Selected DIA1-affected phosphoproteins

Given the secretory pathway localisation of DIA1, it is noteworthy that a number of proteins with phosphosites affected by different levels of DIA1 are located in the Golgi and in the ER. One of these proteins (COPA) is annotated as involved in ER to Golgi vesicle-mediated transport and also in the opposite process of retrograde vesicle-mediated transport, Golgi to ER. DIA1 was previously shown to colocalise with related beta-coatomer proteins (Takatalo et al., 2009).

When analysing proteins with phosphosites dependent on DIA1, we considered some cases with borderline significance (*t*-test *p*-value < 0.05 and FDR *q*-value < 0.2) if the proteins had ER or Golgi localisation. One such protein whose phosphorylation is borderline affected by DIA1, calnexin (*p*-value 0.03, *q*-value 0.18), is a multifunctional molecule, serving as a molecular chaperone of the ER (Chevet et al., 2010). Phosphorylation at conserved sites (S534, S544) has been ascribed to CK2 and regulates distribution of calnexin on ER (Chevet et al., 2010; Wong et al., 1998). Our results suggest that calnexin phosphorylation at S534 and S544 may be regulated by DIA1.

Also, upon DIA1 expression, the phosphorylation of S303 in the SCFD1 (Sec1 family domain-containing protein 1, Sly1p) borderline increases (*p*-value 0.02, *q*-value 0.16). SCFD1 is involved in vesicle fusion, in the context of vesicular transport between the ER and the Golgi (Demircioglu, Burkhardt & Fasshauer, 2014). Phosphorylation of S303, located on the protein surface and away from the syntaxin binding site (Bracher & Weissenhorn, 2002), may affect SCFD1 interactions with other elements of the vesicle fusion machinery.

Another protein where phosphorylation increased significantly in the presence of DIA1 is the GTP-binding protein SAR1A. This important component of the coat protein II (COPII) is a trafficking protein that modulates membrane rigidity. SAR1A initiates formation of COPII vesicles and thus regulates ER to Golgi transport (Brandizzi & Barlowe, 2013; Loftus, Hsieh & Parthasarathy, 2012).

Further, phosphorylation of a phosphosite in GORASP2 (GRASP55) is increased upon the presence of DIA1. GORASP2, known to be regulated by ERK, is required for Golgi ribbon formation (Feinstein & Linstedt, 2008). The DIA1-dependent site (Thr222) has been reported to be an ERK site (Vinke, Grieve & Rabouille, 2011). Interestingly, GORASP2 is also involved in unconventional secretion, and this role is also phosphorylation-dependent (Kim et al., 2016).

DIA1-overexpressing cells also feature increased phosphorylation of Serine incorporator 1 (SERINC1), an ER serine transporter involved in membrane biogenesis (Inuzuka, Hayakawa & Ingi, 2005). Our results show that in the presence of DIA1 changes occur in phosphorylation of a number of proteins involved in the secretory pathway. However, the diverse phosphorylation site motifs (see Table 1) suggest that the effects of DIA1 on phosphorylation may be indirect.

Three of DIA1-dependent phosphoproteins are kinases, namely the CDK13 (cyclin-dependent kinase 13), TNNI3K (serine/threonine-protein kinase TNNI3K) and TTK (dual specificity protein kinase TTK). The relationships of these kinases with DIA1 are likely indirect, however, because their localisation is not consistent with the localisation of DIA1.

The strong effect of the presence of DIA1 on CK2 phosphosites is interesting in the context of CK2 roles in the development of the nervous system and its status as a promising neurological drug target (Castello et al., 2017; Litchfield, 2003). However, it cannot be ruled out that the observed changes in CK2 sites are partly due to activities of other kinases. The phosphosites of casein kinase 2 (CK2) and those of Golgi casein kinase (FAM20C/G-CK) are rather similar (SxE and S/Txx[ED], respectively) (Tagliabracci et al., 2014). Current bioinformatics tools and databases for phosphosite analysis (e.g. KinomeXplorer) may not be able to distinguish between these two kinds of sites (Lasa-Benito et al., 1996; Tibaldi et al., 2015). For example it has been shown that in the CK2-null cells only less than one-third of ‘CK2’ phosphosites are significantly decreased (Franchin et al., 2017). Therefore, our results do not allow the hypothetical direct phosphorylation by DIA1 to be distinguished from its indirect effects, related or not to the hypothetical kinase activity.

Despite very strong evolutionary conservation of the predicted kinase active site in the DIA1 family (Dudkiewicz, Lenart & Pawlowski, 2013), it cannot be excluded that DIA1 lacks enzymatic activity altogether, and is a pseudokinase, one of an emerging class of kinase-like proteins with non-catalytic functions in cell signalling (Jacobsen & Murphy, 2017). Although DIA1 is the official the gene symbol for the Deleted in Autism 1, the gene symbol DIA1 has also previously been used for two other human genes: DIAPH1 (diaphanous related formin 1) and CYB5R3 (cytochrome b5 reductase 3).

DIA1 has recently been characterised as a secreted paracrine factor and ligand for IGF1R (Bareja et al., 2017). It has been found to promote cardiomyocyte regeneration via PI3K kinase by stimulating proliferation and via PKCε by blocking apoptosis (Beigi et al., 2013; Huang et al., 2014; Miura, 2014). These activities might occur via DIA1-mediated phosphorylation of extracellular domains of IGF1R, and indeed the PhosphositePlus database lists six such sites, although none was observed in this experiment. However, DIA1 signalling via IGF1R might be independent of any kinase activity on its part. The effects described by Dzau and co-workers and by us might be an effect of a growth-factor-like function for a kinase-like protein.

The fact that no very strong effects of DIA1 expression on phosphopeptide abundance are observed here may suggest that it is a pseudoenzyme. However, the strong evolutionary conservation of the predicted active site suggests otherwise, and DIA1 may require an as yet undiscovered activation mechanism to achieve its optimal enzymatic capability. Further investigation into the molecular roles of this intriguing protein may require massive effort combining phosphoproteomics, genetics and pharmacology in a manner similar to the LRRK2 study (Steger et al., 2016) and may also require the inclusion of other family members, namely FAM69A, B and C as well as DIA1R. If DIA1 is a pseudokinase, then it may well act as a modulator of a related active kinase, similarly to the FAM20C/FAM20A kinase/pseudokinase pair reported recently to act in the secretory pathway (Cui et al., 2015).

## Conclusion

Only one in five known phosphosites are mapped to a kinase. Moreover, the potential regulatory functions of most phosphosites have not been revealed (Munk, Refsgaard & Olsen, 2016; Munk et al., 2016).

Here, we have shown in an in vitro model that the expression of the predicted kinase DIA1 is related to changes in many phosphosites in different cellular compartments. Although direct and indirect effects of DIA1 could not be separated in this study, it is likely that DIA1 positively regulates CK2 signalling and also signalling by a proline-directed kinase. It cannot be excluded that a different kinase with CK2-like consensus is involved, or that DIA1 itself has a CK2-like substrate motif. Nevertheless, the kinase activity of DIA1 still remains to be proven or disproven. The detailed elucidation of enzymatic or other activities of DIA1 and its signalling network will require further studies in different cellular systems.

## Supplemental Information

10.7717/peerj.4599/supp-1 Supplemental Information 1 Pearson correlation for global protein analysis and phosphorylation analysis. Multiscatter plots with calculated Pearson correlation for global protein analysis (A) and phosphorylation analysis (B) calculated between all replicates in both experimental groups. The presented results were obtained for normalized data.

10.7717/peerj.4599/supp-2 Supplemental Information 2 Protein identity overlap before and after enrichment. Venn diagrams representing number of non-phosphorylated (wild-type group–A, DIA1 overexpressing group–C) and phosphorylated proteins (wild-type group–B, DIA1 overexpressing group–D) identified in at least four replicates in non-enriched and phospho-enriched samples.

10.7717/peerj.4599/supp-3 Supplemental Information 3 Uncropped blot images for Fig. 2.

10.7717/peerj.4599/supp-4 Supplemental Information 4 Phosphosite comparative analysis: DIA1 vs control. MaxQuant results for the phosphosite comparative analysis between DIA1-overexpressing and control cells.

10.7717/peerj.4599/supp-5 Supplemental Information 5 DIA1 interactome. Comparative analysis, DIA1 vs control. MaxQuant results for the proteomic comparative analysis between DIA1-overexpressing and control samples.

10.7717/peerj.4599/supp-6 Supplemental Information 6 Mass Spectrometry technical parameters. Technical parameters of the mass spectrometry experiments and phosphosite statistics.

## Additional Information and Declarations

### Data Availability

The following information was supplied regarding data availability: The mass spectrometry proteomics data are available in the PRIDE database with the dataset identifier PXD006831.
