# Supplementary figures and images for: Phosphoproteomic insights into processes influenced by the kinase-like protein DIA1/C3orf58
Source: PeerJ. 2018 Apr 9;6:e4599. doi: 10.7717/peerj.4599 (PMC5896498; doi:10.7717/peerj.4599)

## B phosphorylation analysis

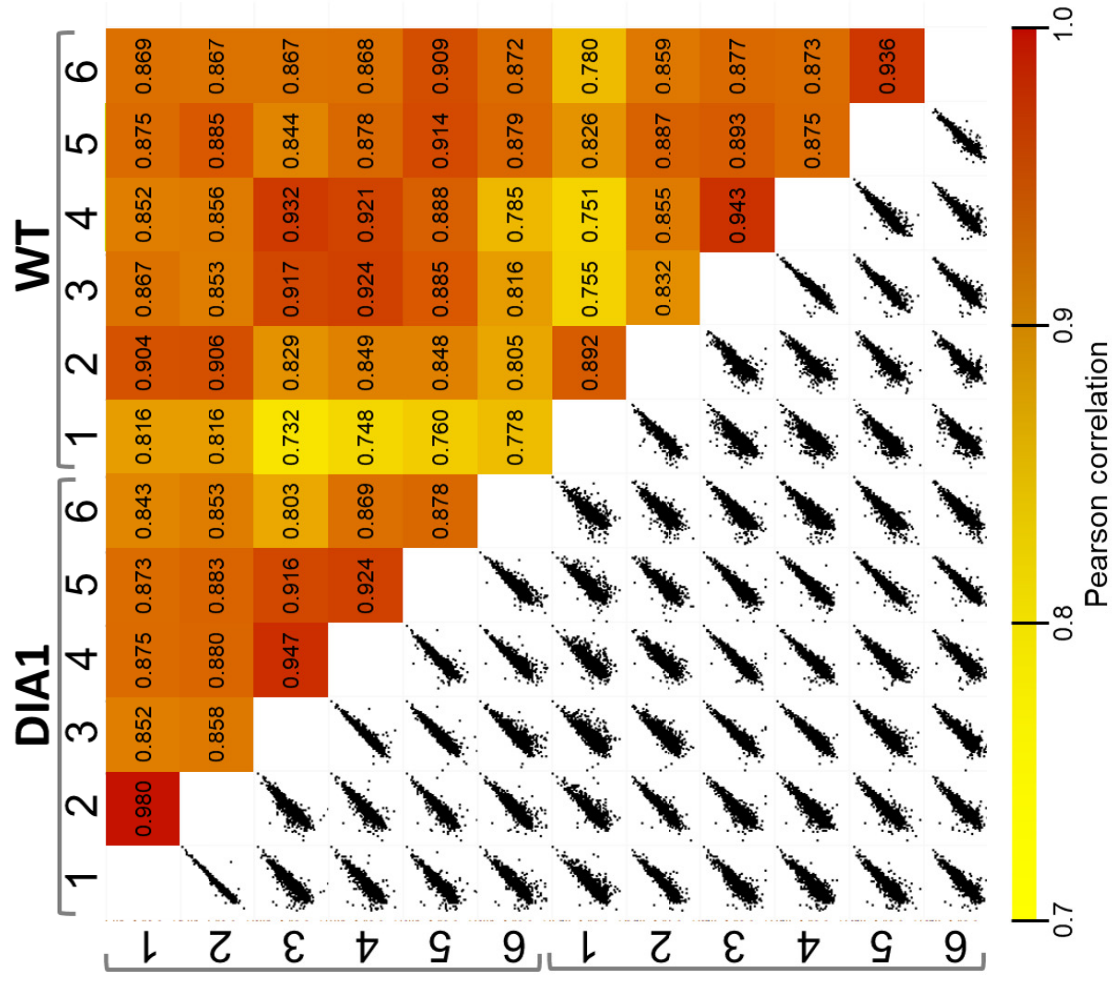

Supplement: Supplemental Information 1 — Multiscatter plots with calculated Pearson correlation for global protein analysis (A) and phosphorylation analysis (B) calculated between all replicates in both experimental groups. The presented results were obtained for normalized data. [file peerj-06-4599-s001.pdf]

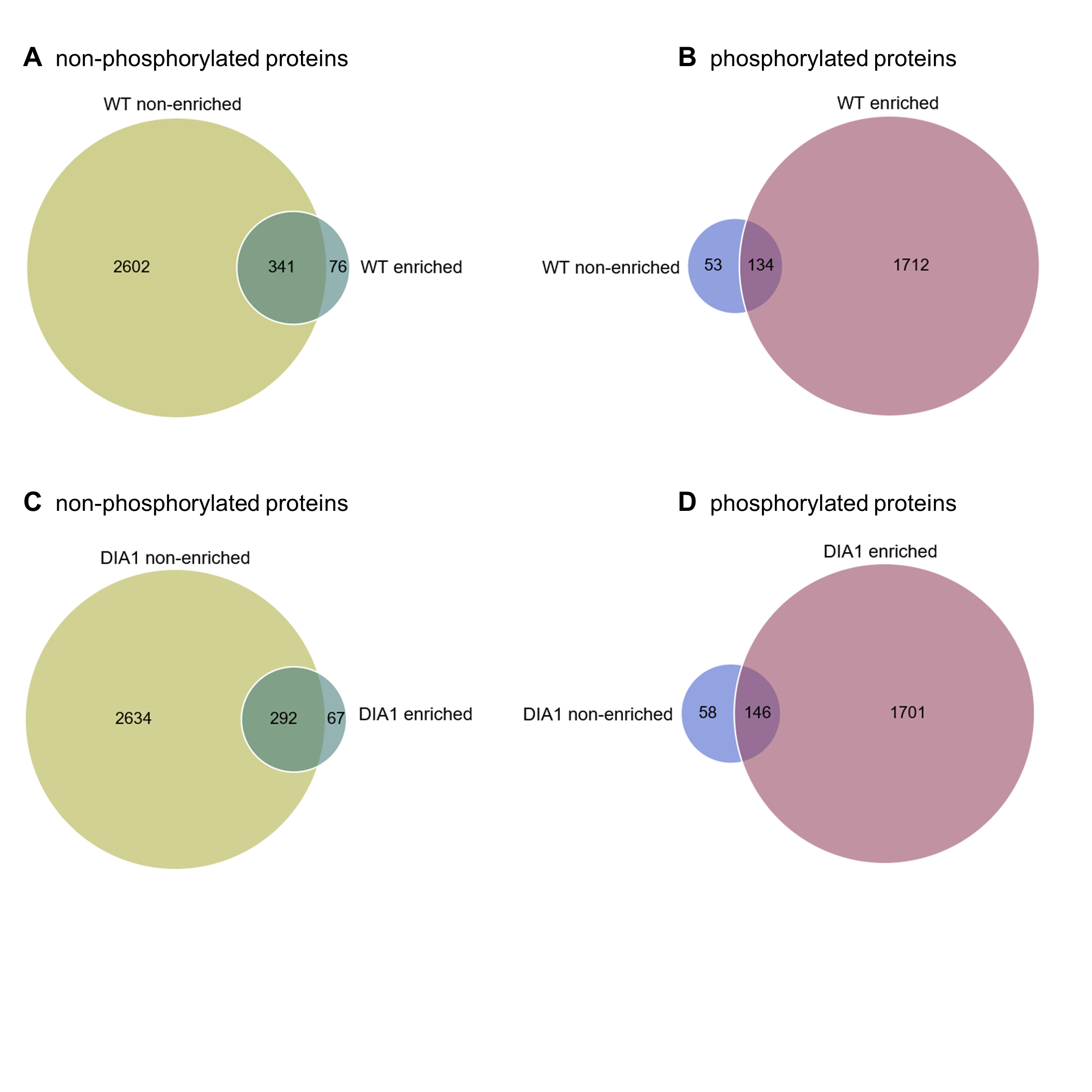

Supplement: Supplemental Information 2 — Venn diagrams representing number of non-phosphorylated (wild-type group–A, DIA1 overexpressing group–C) and phosphorylated proteins (wild-type group–B, DIA1 overexpressing group–D) identified in at least four replicates in non-enriched and phospho-enriched samples. [file peerj-06-4599-s002.png]

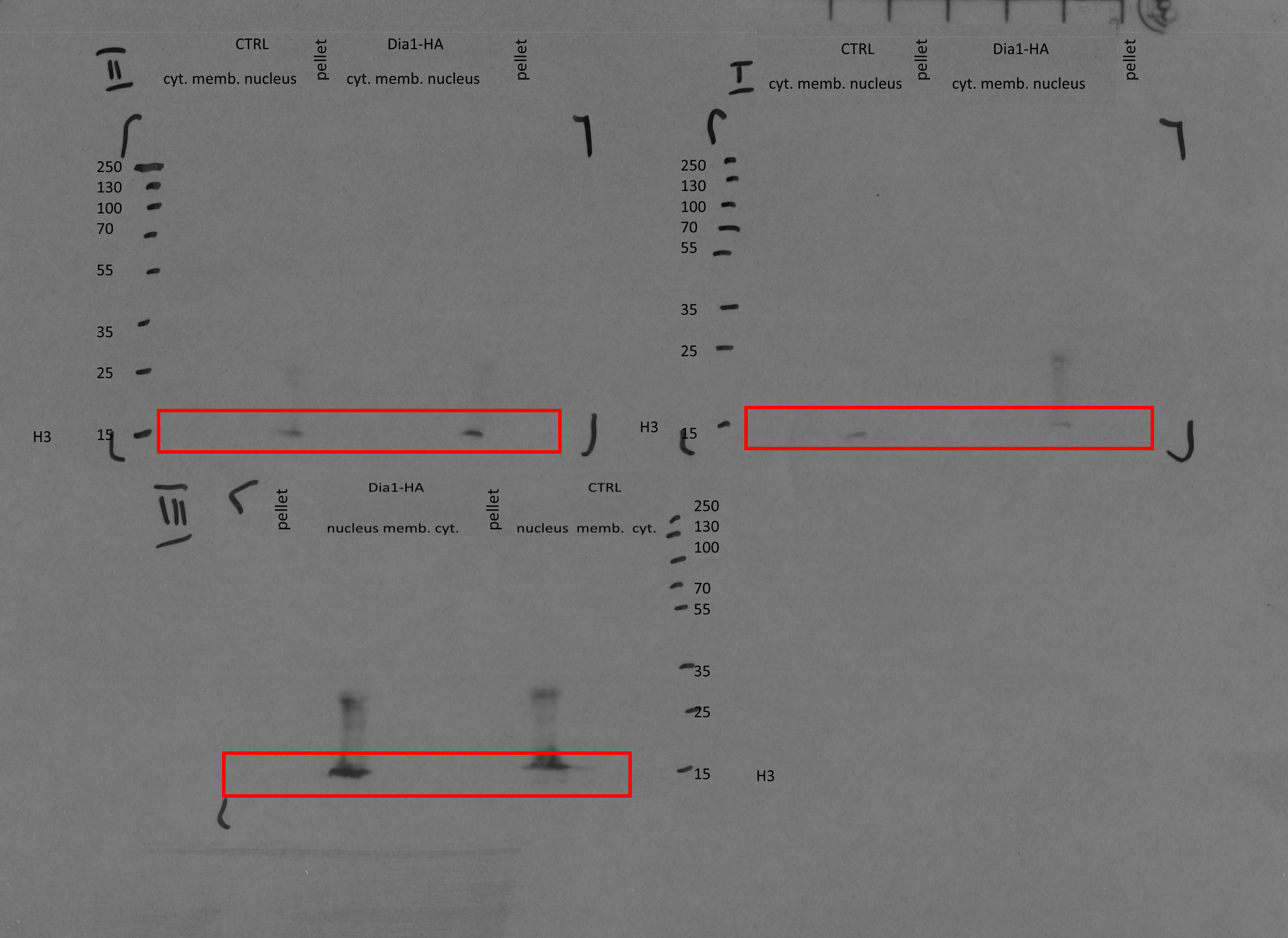

Supplement: Supplemental Information 3 [file peerj-06-4599-s003.zip › histon3_corrected.tif]

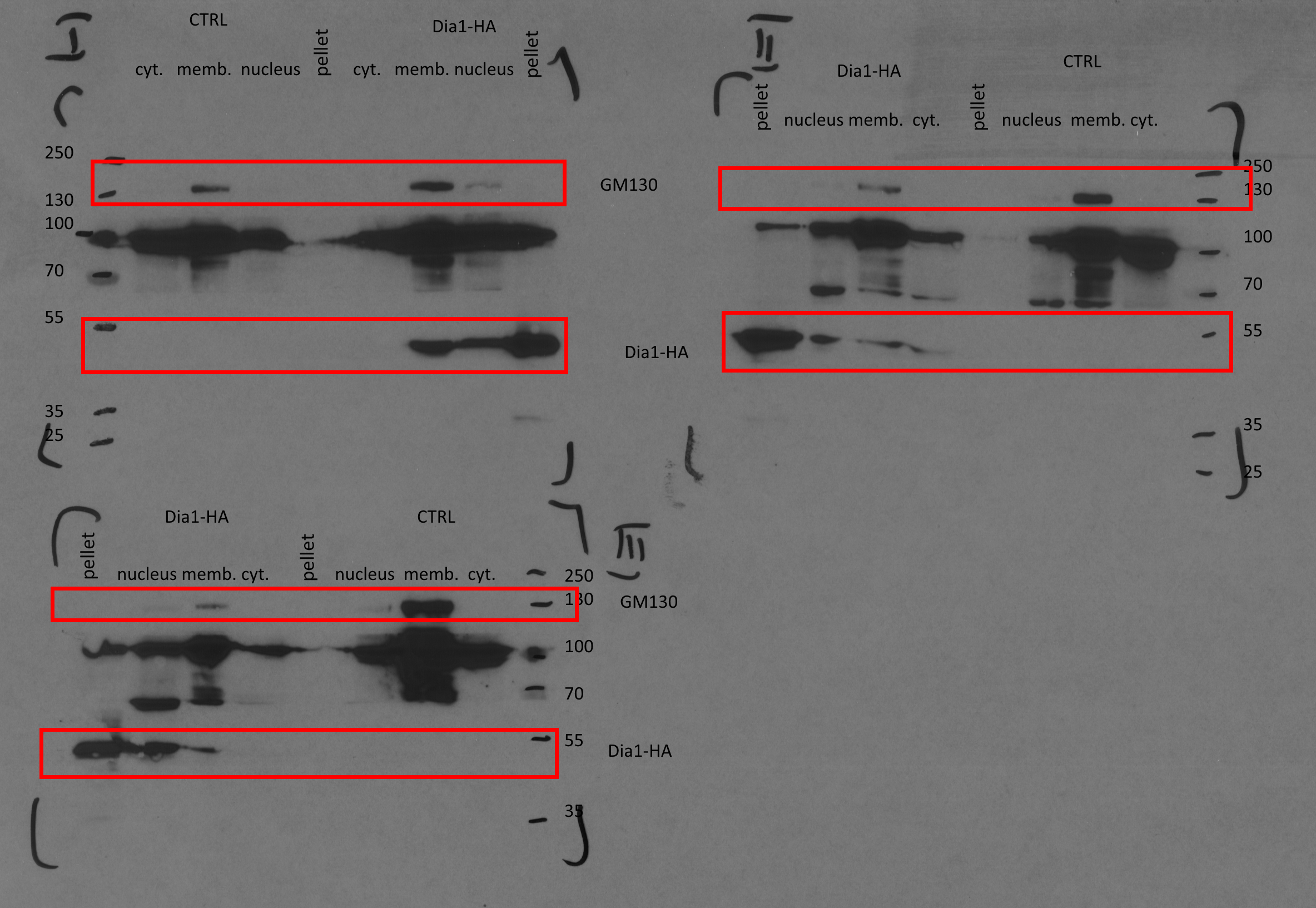

Supplement: Supplemental Information 3 [file peerj-06-4599-s003.zip › GM130 Dia1HA-corrected.tif]

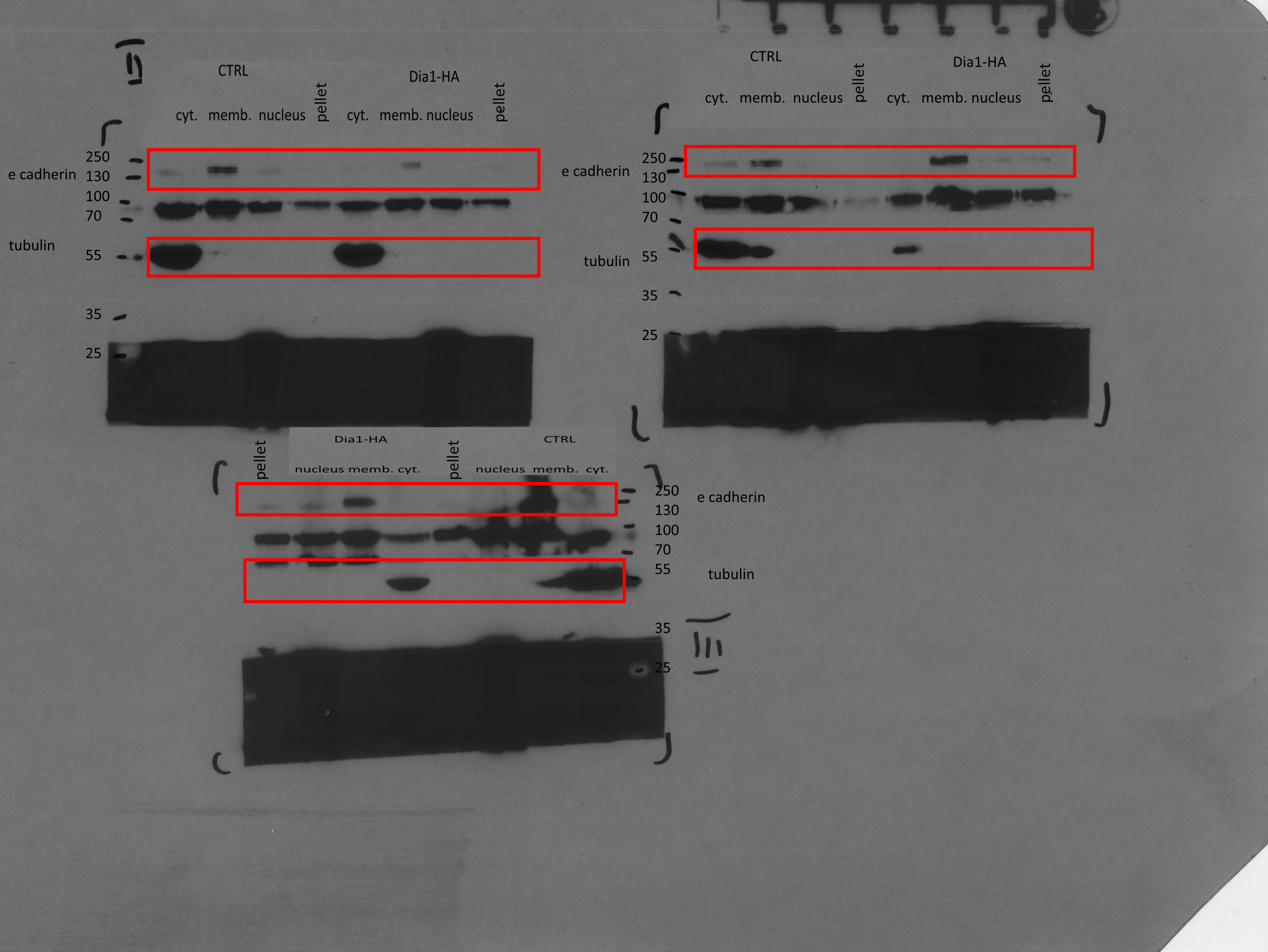

Supplement: Supplemental Information 3 [file peerj-06-4599-s003.zip › tubulin ecadherin-corrected.tif]
